# Deep Small RNA Sequencing of *BRAF* V600E Mutated Papillary Thyroid Carcinoma With Lymph Node Metastasis

**DOI:** 10.3389/fgene.2019.00941

**Published:** 2019-10-08

**Authors:** Azliana Mohamad Yusof, Rahman Jamal, Sazuita Saidin, Rohaizak Muhammad, Shahrun Niza Abdullah Suhaimi, Isa Mohamed Rose, Wan Fahmi Wan Nazarie, Francis Tieng Yew Fu, Nurul-Syakima Ab Mutalib

**Affiliations:** ^1^Cytogenetics and Molecular Diagnostics Laboratory, Pantai Premier Pathology Sdn Bhd, Kuala Lumpur, Malaysia; ^2^UKM Medical Molecular Biology Institute (UMBI), Universiti Kebangsaan Malaysia, Kuala Lumpur, Malaysia; ^3^Department of Surgery, Faculty of Medicine, Universiti Kebangsaan Malaysia, Kuala Lumpur, Malaysia; ^4^Department of Pathology, Faculty of Medicine, Universiti Kebangsaan Malaysia, Kuala Lumpur, Malaysia; ^5^Institute of Bioscience (IBS), Universiti Putra Malaysia, UPM Serdang, Selangor, Malaysia

**Keywords:** small RNA sequencing, papillary thyroid cancer, lymph node metastasis, BRAF V600E, microRNAs, Piwi-interacting RNA

## Introduction

Papillary thyroid carcinoma (PTC) is the most predominant subtype of thyroid cancer, contributing to more than 80% of all thyroid or endocrine malignancies. Its prognosis is relatively good compared to other cancers, with more than 90% overall 10-year survival rate (Haugen et al., 2016). However, despite the favorable prognosis, some cases exhibit aggressive phenotype. On average, 50% of the patients are presented with lymph node metastases (LNM) at diagnosis (Saiselet et al., 2015). LNM in PTC confers various poor prognostic indicators; it increases recurrence risk and decreases long term survival predominantly in patients older than 45 years old (Lundgren et al., 2006; Xing, 2013). Treating recurrent PTCs is still a challenge (Pasternak and Shen, 2017) and one of the important issues yet to be solved in PTC patient management is the mortality and morbidity associated with the recurrent disease (Lee et al., 2013).

MicroRNAs (miRNAs) are short, endogenous noncoding RNAs first identified in *Caenorhabditis elegans* which regulate gene expression by binding to the 3′-UTR of target messenger RNA (mRNAs). Their post-transcriptional regulatory functions are involved in controlling the level of proteins involved in numerous biological processes, including embryogenesis, organogenesis, tissue homeostasis, immune system function and cell cycle control (Tafrihi and Hasheminasab, 2019). Relationship between miRNAs and tumor growth, tumor progression and metastasis has been demonstrated by many studies, indicating the ability of these molecules to be used as biomarkers for diagnosis and prognosis (Iorio and Croce, 2012). MiRNA also plays a role as biomarker in predicting lymph nodes metastasis (LNM) in thyroid cancer (Ab Mutalib et al., 2016; Mutalib et al., 2016; Mohamad Yusof et al., 2018).

P-element-induced wimpy testis (PIWI)-interacting RNAs (piRNAs) are a quite recently discovered class of RNAs and more than 30,000 piRNA genes been identified within the human genome (Zhang et al., 2014; Wang et al., 2019). Originally described as key regulators for the germline maintenance and transposon silencing, these short RNAs were disregarded for a long time due to our limited knowledge regarding their function (Weng et al., 2019). However, emergent of new data reveals that unusual expression of piRNAs is a distinct feature Fin many diseases, including cancers (Weng et al., 2019). This has changed our perspectives on their significance in various diseases. Epigenetically, piRNAs are also the post-transcriptional regulators of the expression of specific downstream target genes (Watanabe and Lin, 2014), and accumulating evidence demonstrates that similarly to miRNAs, piRNAs also possess both oncogenic and tumor-suppressive roles (Weng et al., 2019).

BRAF is a serine-threonine kinase that is activated by RAS binding and protein recruitment to the cell membrane. Activation of MEK along with the MAPK signaling pathway is activated by BRAF phosphorylation (Nikiforov and Nikiforova, 2011). The most frequent genetic changes in PTC are point mutations of *BRAF* which are observed in 35 to 70% of PTC cases (Li et al., 2012; Xing, 2013). More than 95% of *BRAF* mutations in thyroid cancers are thymine to adenine transversion at position 1799 (T1799A) which result in the substitution of glutamate from valine at residue 600 (V600E) (Xing et al., 2015). Various studies have also shown that the *BRAF* V600E mutation is related to LNM, as further presented by a meta-analysis by Song et al. (2018).

In the past years, the evolution of next-generation sequencing technologies has enabled global transcriptome expression profiling and the discovery of novel human miRNAs (Kozomara and Griffiths-Jones, 2011; Kozomara and Griffiths-Jones, 2014; Kozomara et al., 2019) and piRNAs (Weng et al., 2019). Coincidentally, PTC has a relatively lower overall mutation burden hence termed as quiet genome (Cancer Genome Atlas Research Network, 2014; Siraj et al., 2016), therefore investigating the regulatory molecules such as the miRNAs and piRNAs has the potential to deepen our understanding of this cancer. This Data Report aims to provide the readers a comprehensive dataset of small RNAs expression derived from next-generation sequencing in an unbiased manner. For detailed analysis of this dataset focusing on miRNAs with biological insights in PTC, please refer to our original article published in Frontiers of Endocrinology (Mohamad Yusof et al., 2018).

## Methods

### Clinical Specimens

**Figure 1:**
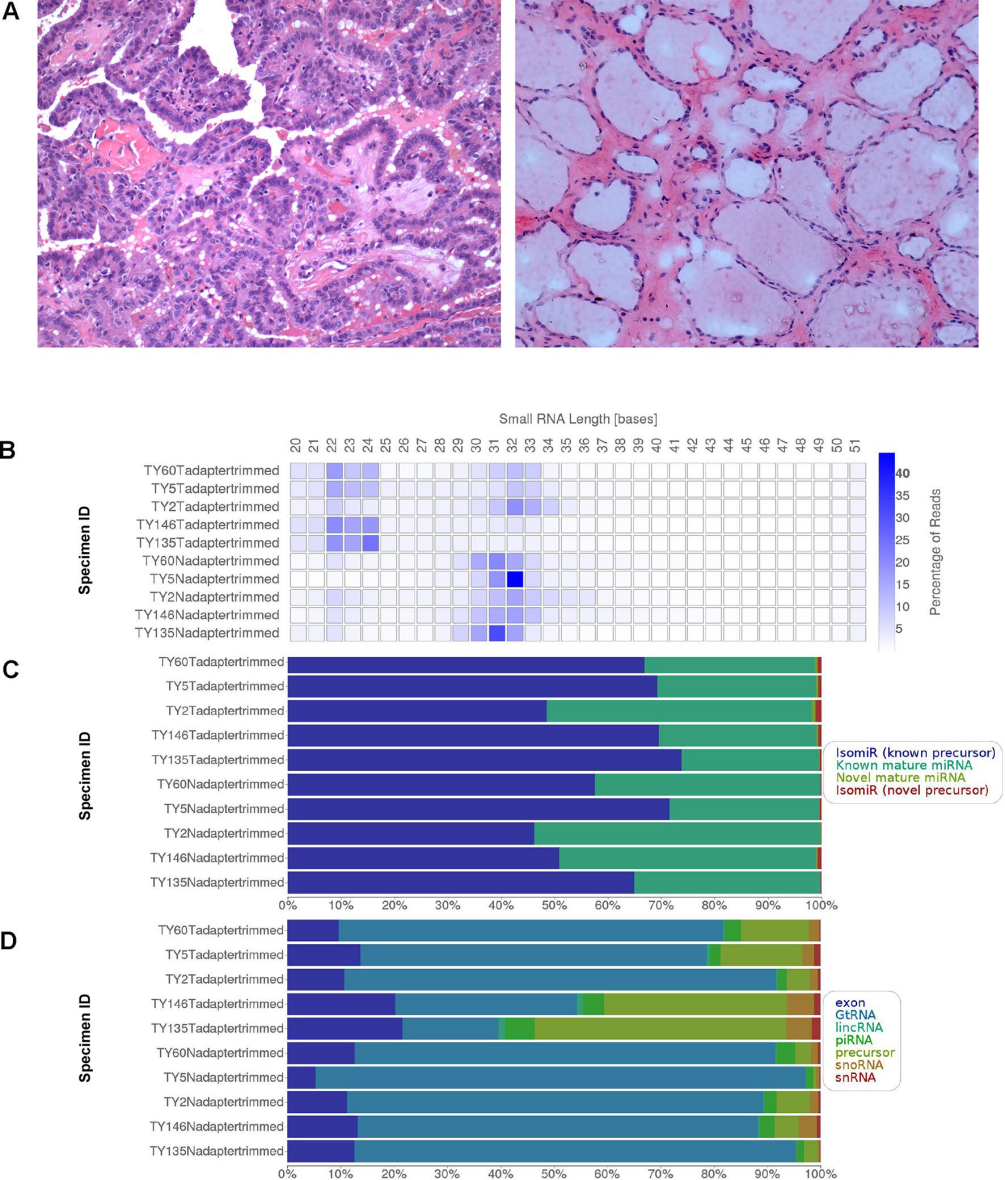
**(A)** Representative H&E staining of the PTC (left) and adjacent normal thyroid (right) under 200× magnification. **(B)** Small RNA length distribution **(C)** miRNA hits by category and **(D)** other RNA hits by category.

Five pairs of tumor-adjacent normal fresh frozen thyroid tissues (n = 10) were collected from PTC patients with lymph node metastasis (N stage = N1, N1a or N1b) from the UKM Medical Centre (UKMMC). Only the thyroid specimens were collected and there was no metastatic lymph node included for several reasons. Firstly, we would like to study the source of the metastasis, which is from the cancer cells at the primary origin (the thyroid itself). Another reason was that the metastatic lymph nodes were unavailable from our Biobank. The resected lymph nodes were all sent to the Pathology Laboratory for diagnostic confirmation. Henceforth, the nodes are fixed in formalin which renders the specimens unsuitable for next-generation sequencing experiment due to RNA degradation. This study was approved by the Universiti Kebangsaan Malaysia Research Ethics Committee (UKMREC; UKM 1.5.3.5/244/UMBI-2015-002). Informed consent was obtained from all the study participants. The tissues were dissected, snap-frozen and stored in liquid nitrogen. All samples were cryosectioned and stained with hematoxylin and eosin and the percentage of tumor cells and normal cells contents were confirmed by a pathologist. The representative figures of the staining at 200× magnification were provided in **Figure 1A** (left: PTC, right: normal adjacent thyroid). Only tumor samples with at least 80% cancerous cells and normal adjacent thyroid tissues with less than 20% necrosis were selected for small RNA sequencing. The tissues were subjected to nucleic acids extraction using Allprep DNA/RNA/miRNA Universal Kit (Qiagen, Valencia, CA, USA) according to manufacturer’s recommendations. The integrity of RNA was assessed using Agilent Bioanalyzer 2100 (Agilent Technologies, Santa Clara, CA, USA) while the quantity and purity of RNA were assessed using Qubit 2.0 fluorometer and Nanodrop 2000c Spectrometer (Thermo Fisher Scientific, Pittsburgh, PA, USA), respectively.

### *BRAF* V600E Genotyping

*BRAF* V600E genotyping was performed using forward primer 5′-TGCTTGCTCTGATAGGAAAATG-3′ and reverse primer 5′-AGCATCTCAGGGCCAAAAAT-3′ (Monti et al., 2015). The DNA sequencing was performed on ABI Prism 3130xl Genetic Analyzer (Applied Biosystem, USA).

### Libraries Preparation

RNA from tumor and their adjacent normal tissues were processed into libraries using TruSeq Small RNA Sample Prep Kit (Illumina, San Diego, CA, USA). Briefly, 3′ and 5′ adapters were sequentially ligated to the ends of small RNAs fractionated from 2 μg of total RNA, and reverse transcribed to generate cDNA. The cDNA was amplified using a common primer complementary to the 3′ adapter, and a primer containing 1 of 48 index sequences. Samples were size-selected (140–160 bp fragments) on a 6% polyacrylamide gel, purified, quantified and pooled for multiplexed sequencing.

### Small RNA Sequencing

The resulting pooled libraries were normalized to 2 nM and were hybridized to oligonucleotide-coated single-read flow cells for cluster generation using HiSeq^®^ Rapid SR Cluster Kit v2 on Hiseq 2500 (Illumina, San Diego, CA, USA). Subsequently, the clustered pooled microRNA libraries were sequenced on the HiSeq 2500 for 50 sequencing cycles using HiSeq^®^ Rapid SBS Kit v2 (50 Cycle) (Illumina, San Diego, CA, USA). The sequencing run was completed on December 18, 2015.

### Bioinformatics and Statistical Analyses

Pre- and post-processing of data were executed in BaseSpace software (Illumina, San Diego, CA, USA). Illumina Sequence Integration Software (Isis) version 2.5.52.11 processed raw data from Illumina sequencers into FASTQ files. Subsequently, Small RNA Analysis app version 1.0.0.0 was used for determination of differentially expressed small RNAs. Differential expression analysis is performed for the following marker types; miRNA family, precursor group, miRNAs (include mature miRNAs and isomiRs) and piRNAs. Briefly, this workflow uses Bowtie version 0.12.8 to align each cluster against abundant, mature miRNA, other RNA, and genomic reference databases (Langmead et al., 2009). The reference genome used for the alignment was Homo sapiens maskedPAR (UCSC hg19). The output from Bowtie was then processed by SAMtools 0.1.19-isis-1.0.2 (Li et al., 2009), annotated to miRBase v21 (Kozomara and Griffiths-Jones, 2014; Kozomara et al., 2019) and performed differential analysis using DESeq2 (Love et al., 2014). DESeq2 removed low expressed markers with normalized mean count < 10 before testing. The DESeq2 variance model is used to detect and exclude outliers based on the extreme variation between replicates. The status (filtered or passed) and the result of the analysis (mean expression, fold change, standard error, p-value, q-value, etc.) were reported for each marker and will be available upon request. Additionally, novel miRNA and precursor discovery are performed for each sample group using miRDeep* version 3.2 (An et al., 2013).

## Description of Analysis Output

### Small RNA Sequencing Yield

The deep sequencing of small RNA libraries derived from PTC tissues and adjacent normal thyroid yielded 130,459,076 reads with 122,182,874 reads passing filters (93.3%) and 96.25% reads had Phred score of ≥Q30. The total reads that passed filter per sample range from 8,331,660 to 16,629,882 reads, generating comprehensive digital profiles of small RNAs expression in PTC. Summary of small RNA sequencing results is provided in **Table 1**. **Figure 1** illustrates the small RNA length distribution, miRNA hits by category and other RNA hits by category. In the cancer specimens, the size distribution was mainly 22–24 bases and/or 30–34 bases (**Figure 1B**). Interestingly, the size distribution of small RNA in normal thyroid appeared bigger and more consistent, which is in the range of 30 to 33 bases. Focusing on only the miRNAs, our alignment resulted in average more than 50% of the reads were mapped to isomiR of known precursors, followed by known mature miRNAs (**Figure 1C**). Additionally, small percentage of the reads were mapped to the novel mature miRNAs and isomiRs from novel precursor. In **Figure 1D**, we present the outcome of the alignment from other coding and noncoding RNA species, including the exonic RNAs, genomic transfer RNAs (GtRNA), long intergenic noncoding RNAs (lincRNA), piRNAs, precursors, small nucleolar RNAs (snoRNAs) and small nuclear RNAs (snRNAs). The lincRNAs gained wide attention in cancer research lately and with our deep sequencing data, the expression profiles of lincRNAs can be subjected to future research. Meanwhile, the roles of GtRNA, snoRNA and snRNA in cancers are also emerging.

**Table 1:** Summary of small RNA sequencing results.

	Known	Novel	Total
**miRNAs**			
Total miRNAs	4,662	173	4,835
Tested miRNAs	1,079	26	1,105
Differentially expressed miRNAs	252	16	268
**Precursors**			
Total Precursor Groups	971	95	1,066
Tested Precursor Groups	219	36	255
Differentially Expressed Precursor Groups	58	16	74
**miRNA families**			
Total miRNA Families	418	0	418
Tested miRNA Families	150	0	150
Differentially expressed miRNA families	36	0	36
**piRNAs**			
Total piRNAs	848	0	848
Tested piRNAs	110	0	110
Differentially expressed piRNAs	19	0	19

Tested markers are those that pass the low-count filter and are tested for statistical significance. Novel markers (when applicable) are predicted by miRDeep*.

### Mature miRNAs and isomiRs

Expression profiles for 4,662 previously annotated miRNAs were delineated and 1,079 miRNAs passed the filter and subjected to differential analysis. A set of 252 miRNAs were significantly differentially expressed (q value < 0.05) in cancer versus normal thyroid tissues. There were 134 upregulated miRNAs and 118 downregulated miRNAs.

### Novel miRNAs

Expression profiles for 173 novel miRNAs predicted by miRDeep* were described; 26 novel miRNAs passed the filter and subjected to differential analysis. Sixteen novel miRNAs were significantly differentially expressed (adjusted p-value < 0.05) in cancer versus normal thyroid tissues; 11 novel miRNAs were upregulated while five were downregulated.

### Precursor Group

There were 971 precursors detected in this dataset, with 219 precursors passed the filter and subjected to differential analysis. A set of 58 precursor miRNAs were significantly differentially expressed (q value < 0.05) in cancer versus normal thyroid tissues. There were 33 upregulated and 25 downregulated precursor miRNAs, respectively.

### Novel Precursor miRNAs

There were 95 novel precursors predicted from this dataset, with 36 novel precursors passed the filter and subjected to differential analysis. A set of 16 novel precursor miRNAs were significantly differentially expressed (q value < 0.05) in cancer versus normal thyroid tissues. There were 4 upregulated and 12 downregulated novel precursor miRNAs, respectively.

### miRNA Family

Expression profiles for 418 previously annotated miRNA families were detected and 150 families passed the filter and subjected to differential analysis. A set of 36 miRNA families were significantly differentially expressed (q value < 0.05) in cancer versus normal thyroid tissues. There were 17 upregulated and 19 downregulated miRNA families, respectively. The workflow does not predict novel miRNA families.

### piRNA

Expression profiles for 848 previously annotated piRNAs were detected and 110 piRNAs passed the filter and subjected to differential analysis. A set of 19 piRNAs were significantly differentially expressed (q value < 0.05) in cancer versus normal thyroid tissues. There were 8 upregulated piRNAs and 11 downregulated piRNAs. The workflow does not predict novel piRNAs.

### Direct Link to Deposited Data and Information to Users

The small RNA sequencing data in fastq. format was deposited at the NCBI Sequence Read Archive (SRA) at http://www.ncbi.nlm.nih.gov/sra with accession number PRJNA378313 (SRP101463). The sample IDs are TY135, TY 146, TY2, TY5 and TY60. This raw data will enable other researchers to perform mapping and differential expression analysis according to their specific objectives.

## Data Availability Statement

The datasets generated for this study can be found in the NCBI Sequence Read Archive (SRA), PRJNA378313 (SRP101463).

## Ethics Statement

The studies involving human participants were reviewed and approved by Universiti Kebangsaan Malaysia Research Ethics Committee. The patients/participants provided their written informed consent to participate in this study.

## Author Contributions

AMY and N-SAM involved in the specimen collections, libraries preparation and sequencing, data analyses, acquisition of data and drafting the manuscript. RJ provides a critical review of the manuscript. SS performed *BRAF* V600E genotyping, cryosectioning and staining of the fresh frozen tissues. RM and SNAS are thyroid surgeons involved in specimen retrieval. IMR assessed tumor percentage of the tissues. FTYF was involved in the initial draft. WFWN is the bioinformatician partly involved in data analysis. All authors read and approved the final manuscript.

## Funding

This research was funded by the Fundamental Research Grant Scheme (FRGS) from the Ministry of Education Malaysia (FRGS/1/2014/SKK01/UKM/03/1).

## Conflict of Interest

The authors declare that the research was conducted in the absence of any commercial or financial relationships that could be construed as a potential conflict of interest.
